# Regulation of HIV self‐testing in Malawi, Zambia and Zimbabwe: a qualitative study with key stakeholders

**DOI:** 10.1002/jia2.25229

**Published:** 2019-03-25

**Authors:** Russell J Dacombe, Victoria Watson, Lot Nyirenda, Claudius Madanhire, Musonda Simwinga, Lignet Chepuka, Cheryl C Johnson, Elizabeth L Corbett, Karin Hatzold, Miriam Taegtmeyer

**Affiliations:** ^1^ Community Health Systems Group Department of International Public Health Liverpool School of Tropical Medicine Liverpool UK; ^2^ Centre for Sexual Health HIV and AIDS Research Harare Zimbabwe; ^3^ Zambart Lusaka Zambia; ^4^ Medical and Surgical Nursing Department Kamuzu College of Nursing Blantyre Malawi; ^5^ HIV Department World Health Organization Geneva Switzerland; ^6^ Clinical Research Programme Malawi Liverpool Welcome Trust Blantyre Malawi; ^7^ Population Services International Harae Zimbabwe

**Keywords:** quality assurance, policy, *in vitro* diagnostics, post market, implementation, harmonization

## Abstract

**Introduction:**

HIV self‐testing (HIVST) is being introduced as a new way for more undiagnosed people to know their HIV status. As countries start to implement HIVST, assuring the quality and regulating *in vitro* diagnostics, including HIVST, are essential. We aimed to document the emerging regulatory landscape and perceptions of key stakeholders involved in HIVST policy and regulation prior to implementation in three low‐ and middle‐income countries.

**Methods:**

Between April and August 2016, we conducted semi‐structured interviews in Malawi, Zambia and Zimbabwe to understand the relationships between different stakeholders on their perceptions of current and future HIVST regulation and the potential impact on implementation. We purposively sampled and interviewed 66 national‐level key stakeholders from the Ministry of Health and the regulatory, laboratory, logistical, donor and non‐governmental sectors. We used a thematic approach to analysis with an inductively developed common coding framework to allow inter‐country comparison of emerging themes.

**Results:**

In all countries, the national reference laboratory was monitoring the quality of HIVST kits entering the public sector. In Malawi, there was no legal mandate to regulate medical devices, in Zambia one regulatory body with a clear mandate had started developing regulations and in Zimbabwe the mandate to regulate was overlapping between two bodies. Stakeholders indicated that they had a poor understanding of the process and requirements for HIVST regulation, as well as lack of clarity and coordination between organizational roles. The need for good collaboration between sectors, a strong post‐market surveillance model for HIVST and technical assistance to develop regulators capacity was noted as priorities. Key informants identified technical working groups as a potential way collaboration could be improved upon to accelerate the regulation of HIVST.

**Conclusion:**

Regulation of *in vitro* diagnostic devices, including HIVST, is now being recognized as important by regulators after a regional focus on pharmaceuticals. HIVST is providing an opportunity for each country to develop similar regulations to others in the region leading to a more coherent regulatory environment for the introduction of new devices.

## 1 Introduction

The World Health Organization (WHO) defines HIV self‐testing (HIVST) as “a process in which a person collects his or her own specimen (oral fluid or blood) and then performs a test and interprets the result, often in a private setting, either alone or with someone he or she trusts” 1. HIVST has been put forward as an innovative tool for reaching the remaining 23% (33% to 12%) of people with HIV who do not know their status 2. The number of HIVST kits accessible through the public sector in Africa is increasing rapidly in response to the global scale‐up 3. The regulation of kits has been identified as an important emerging area to protect the consumer from harm 4.

HIVST kits are classed as *in vitro* diagnostic (IVD), that is tests on specimens taken from the body, and thus are considered medical devices by the International Medical Device Regulation Forum 5. Medical devices are classified according to the hazard the device presents based on its intended use and the expertise of the user and the impact of the result. Due to the potentially severe outcomes of an incorrect result and its use by lay persons, regulators would likely consider HIVST kits as a Class D (highest risk) medical device and therefore subject to the greatest degree of regulation 6. For an HIVST kit to meet the stringent regulatory standards of the International Medical Device Forum, it must not only demonstrate the stability and accuracy required for device registration, but also take into account mechanisms for ensuring the kit performs optimally in the hands of intended users. HIVST kits approved for use by regulatory authorities in high‐income countries, such as the United States Food and Drug Administration, may take several years to evaluate and test performance in their specific population 7, 8. To speed up this process and make the evaluation more focused on low‐ and middle‐income countries, in 2016, the WHO released the technical specifications series for the pre‐qualification (PQ) of HIVST kits 9 and in 2017 the OraQuick® HIV Self‐Test was the first device to be given PQ approval 10.

Surveys of regulation across Africa have identified IVD regulation as a neglected area 11, 12. In the majority of countries, including those with generalized HIV epidemics planning to use the HIVST approach as part of their strategic response, HIVST remains unregulated 4, 12. Many low‐ and middle‐income countries and donors use WHO PQ as a pre‐requisite or substitution for device registration. However, PQ does not cover all monitoring of device performance undertaken once a device is on the market (post‐market surveillance) though an adverse event reporting system is in place 13. For professional use HIV rapid diagnostic tests (RDTs), programmes for external quality assurance (EQA) have been developed for resource‐limited settings to compare testing performance between sites 14. In Africa, EQA programmes are largely run by the tertiary HIV referral laboratories or national reference laboratories and act as a post‐market surveillance system in the absence of or, where available, in collaboration with IVD regulators. These approaches require adaptation to work for HIVST. However, at present many of these EQA programmes are not working due to insufficient funding 15.

We set out to determine the current regulatory status of HIVST in Malawi, Zambia and Zimbabwe and document the perceptions and suggestions of key stakeholders regarding current and future HIVST regulation in each country. These countries had been selected for the Unitaid/PSI HIV Self‐Testing in AfRica (STAR) project based on their high HIV prevalence (Malawi 9.2%, Zimbabwe 13.5% and Zambia 12.4%), established community‐based HIV testing services, availability of data from pilot studies on HIVST and importantly, local government support for HIVST 16, 17, 18, 19. The STAR project aims to catalyse the market for high quality HIVST. Appropriate effective regulation is required to meet this aim.

## 2 Methods

We used qualitative and policy analysis methods to understand the relationships between different stakeholders, their perceptions of current and future regulation and its links to potential scale‐up 20, 21. We sought to document the current understanding and knowledge of HIVST regulation and to explore sensitive areas around how the development of regulation for HIVST can be influenced by context and individual stakeholders. Individual semi‐structured interviews with key informants were conducted at the convenience of the key informants 22. The consolidated criteria for reporting qualitative research were used when preparing this manuscript to ensure all relevant information was included 23.

### 2.1 Selection of study participants

The study took place in Malawi, Zambia and Zimbabwe. We considered stakeholders likely to give in‐depth information on regulation in each country and/or who were likely to play a key role in HIVST scale‐up. We developed lists of participants with input from country research teams using both relevant policy and regulatory documents and local knowledge. We further supplemented this list by snowball sampling.

### 2.2 Data collection

We developed topic guides informed by literature on regulation, global and national policies on HIV testing and HIVST and implementation experience related to HIVST and based on the policy triangle framework. The policy triangle is a framework developed to examine not only the content of policy but also why is it needed (context), the stakeholders involved (actors), and how it is developed and implemented (the process). The topic guides focused on questions considered to be important in HIVST including key informants’ perceptions on the current and future processes for regulation of HIVST, key stakeholders in regulation and policy and their relationships and views on the context of scale‐up of HIVST in each country (Data S1). Additional questions were added iteratively after interim analysis of emerging themes. Participants gave written consent to be interviewed. Interviews were conducted in English between April and August 2016, by RD, VW, LN and CM. Interviews were digitally recorded and emerging themes discussed within the research team to triangulate findings.

### 2.3 Data analysis and trustworthiness

Audio recordings of the interviews were transcribed verbatim and NVivo qualitative data analysis Software (QSR International Pty Ltd. Version 11, 2017) was used to manage the data. VW and RD independently coded a subset of ten transcripts each and then met to determine consensus and minimize inter‐coder variability for quality control purposes 24. A thematic approach for data analysis was used which generated themes inductively based on what emerged from the data 25. In order to ensure trustworthiness, initial analysis was discussed and refined by all the interviewers. Findings were then presented to a wider audience of researchers, regulators, WHO staff and policymakers from the three countries at a STAR consortium meeting in Lusaka in October 2016 and an international HIVST workshop held in Nairobi in March 2017, with the subsequent feedback and discussion further informing the analysis 26.

### 2.4 Ethical considerations

We obtained ethical approval from the Liverpool School of Tropical Medicine(Ref: 15.030, University of Zambia (Ref: 013‐11‐15) and Medical Research Council of Zimbabwe (Ref: MRCZ/A/180) and the Malawian College of Medicine Research Ethics Committee (Ref: P.01/16/1860).

## 3 Results

We purposively sampled a total of 66 national‐level key informants across the three countries (Table 1). Three main themes emerged from the interviews: (1) the limited capacity for IVD regulation, (2) the need for improved coordination for IVD regulation, and (3) a desire for international and regional harmonization. These are summarized in Table 2.

**Table 1:** Key informant characteristics

Participant constituency	Number of participants interviewed		
	Malawi	Zambia	Zimbabwe
Ministry of Health Policymaker	6	3	4
Regulator	3	1	4
Laboratory	4	3	2
Pharmacy/stores	1	1	3
NGOs	3	6	7
WHO/UN	2	2	4
Donors	4	0	3
**Total**	**23**	**16**	**27**

WHO, World Health Organization.

**Table 2:** Main themes emerging from interviews

Theme	Country	Category	Supporting Quote	Source
Limited capacity for IVD regulation	Malawi	No authority with legal mandate for IVD regulation	“In fact, they [parliament] are reviewing their Act [of Parliament] at present to include medical devices”	Malawi KII20
	Zimbabwe	Two authorities considered mandated to regulate IVDs	“No, actually I have just assumed that they do go through MCAZ [Medicines Control Authority of Zimbabwe] but I am not sure. It is very unclear”	Zimbabwe KII23
	All	Support required to develop regulations	“Definitely we have to have the capacity and indeed so in the process of our capacity building, we have to actually develop those skills [in IVD regulation]”	Malawi KII14
The need for improved coordination for IVD regulation	All	Weak coordination between ministries of health, regulators and national reference laboratories	“I think the link is quite weak, we don't really have much interaction”	Malawi KII12
	Zimbabwe and Malawi	Need for greater collaboration by regulator	“Medical devices would be a new thing, that's why probably we are doing the regulations. Perhaps then we cannot be exclusive”	Zimbabwe KII 10
	All	Regulator not part of HIV self‐testing technical working groups/task forces	“At the moment we are in what is called drug and medical supplies [technical working group]”	Malawi KII14
International and regional harmonization	All	WHO pre‐qualification an important mechanism for ensuring the quality of test kits	“For now, we are happy to look at what WHO has recommended as a bare minimum, then we will add additional prerequisites ourselves, but it must have a recommendation from WHO. If they are fully pre‐qualified that's even better”	Zambia KII12
	All	Coordination between countries seen as benefit for developing regulations	“It's a matter of trying to get Malawi at the table to see how other countries are doing so they can set up something similar”	Malawi KII13

IVD*, in vitro* diagnostic.

### 3.1 Limited capacity for IVD regulation

Across all three countries, knowledge and understanding of IVD regulation and HIVST was limited. Few key informants were clear on what regulation for HIVST would entail. Both Zambia and Zimbabwe medicines regulatory authorities (Zambia Medicines Regulatory Authority (ZAMRA) and The Medicines Control Authority of Zimbabwe (MCAZ), respectively) reported that they were starting to develop regulations for IVDs, though not specific to HIVST. While most participants in Zambia identified ZAMRA as having the mandate for IVDs, several others thought HIVST regulation would be handled by the Central Medical Stores or the laboratory technical working group. A small number of laboratory staff thought other authorities would need to be involved, such as the Bureau of Standards.

In Zimbabwe, respondents from two authorities reported that they considered themselves to be mandated to regulate HIVST kits (MCAZ and the Medical Laboratory and Clinical Sciences Council of Zimbabwe (MLCSCZ)). However, at the time of the interviews neither had started regulating HIVST kits and it was unclear to respondents who had the regulatory mandate: “No, actually I have just assumed that they do go through MCAZ [Medicines Control Authority of Zimbabwe] but I am not sure. It is very unclear” (Zimbabwe KII23 Male). The majority of respondents reported it was either the MLCSCZ or MCAZ with an approximately equal proportion suggesting it was both. However, some respondents also mentioned the need to have regulatory approvals from the Health Professions Association and the Standards Association of Zimbabwe.

In Malawi, the majority of policymakers and laboratory staff identified that there were no regulated HIVST kits in Malawi. Most identified the national reference laboratory as the responsible body for regulating IVDs, with a few laboratorians and NGO staff respondents reporting that they thought Pharmacy, Medicines and Poisons Board (PMPB) was responsible for regulating IVDs. While respondents indicated that legal mandates for IVDs regulations were unclear, they were aware of a process to provide more clarity, such as the PMPB seeking the mandate to regulate through an Act of Parliament: “In fact, they [parliament] are reviewing their Act [of Parliament] at present to include medical devices” (Malawi KII20 Male). While most identified the national reference laboratory as responsible for IVDs, the majority of respondents felt that regulations of professional use, as well as HIVST kits, should move to the PMPB in the future: “The issue of regulation is different because the reference laboratory is not a regulator. The Pharmacy, Medicines and Poisons Board is a regulator. That is one of their roles” (Malawi KII18 Female).

Regulators in all countries expressed a need for more support to develop IVD regulations. None of the countries had regulations that entirely covered the regulation of IVDs or any specific guidance on HIVST regulation. In Zambia, regulators said they were focusing on getting guidelines developed for the pre‐market registration of products. In Zimbabwe, they were focusing on import and export regulations. In Malawi, regulators were focused on product registration, but noted that they needed support to develop IVD regulations: “Definitely we have to have the capacity and indeed so in the process of our capacity building, we have to actually develop those skills [in IVD regulation]” (Malawi KII14 Male).

### 3.2 The need for improved coordination for IVD regulation

In all countries, significant potential to support HIVST regulation existed between the Ministry of Health HIV department, national reference laboratory and the IVD regulator. Key informants consistently recognized that links between policymakers, regulators and laboratorians were weak: “I think the link is quite weak, we don't really have much interaction” (Malawi KII12 Male). Some regulatory key informants in Zimbabwe and Malawi reflected that greater collaboration maybe useful considering that medical devices were new: “Medical devices would be a new thing, that's why probably we are doing the regulations. Perhaps then we cannot be exclusive” (Zimbabwe KII10 Female).

The lack of an effective regulation system and of a coordinated approach was a concern for all respondents in all countries. A key concern was the potential entry of unregulated and poor quality HIVST kits into the domestic market and was regarded as a risk for all countries. All key informants in Malawi and Zambia indicated that they had not seen HIVST in the private sector. However, in Zimbabwe the majority of policymakers thought that HIVST was available in the private sector, indicating that regulation was urgently required: “We hear people are already selling, kits are out there” (Zimbabwe KII21 Female).

The quality of HIVST kits, particularly their performance in the hands of intended users, was a concern for the majority of key informants across all countries as illustrated by a respondent from Zambia: “If somebody has a false negative, it could be a real issue because they suddenly don't think they have HIV” (Zambia KII4 Male). There was concern from the majority of key informants around the type of post‐market surveillance model to be used for HIVST as it would not be performed by professionals in facilities. The majority of laboratorians and policymakers across countries were concerned with how to monitor false non‐reactive results: “Most likely we will see the positives in the test facility. [The concern is] The ones who come out with a negative and they don't come to the facility” (Zambia KII17 Female).

Technical working groups with a mandate to focus on HIVST were seen as a way of coordinating the development of policy and regulation. In Malawi, most key informants felt the scale‐up of HIVST should be coordinated by the HIV Testing and Counselling Technical Working Sub‐group and a minority of laboratorians thought it should be coordinated through the laboratory technical working group. Notably, neither group included PMPB. They instead belonged to a different technical working group: “At the moment we are in what is called drug and medical supplies [technical working group]” (Malawi KII14 Male).

In Zambia, there was no regulatory involvement in the HIV counselling and testing technical working groups though one policymaker indicated that ZAMRA initiated some *ad hoc* meetings with the Ministry of Health. Some policymakers indicated that approval by the national reference laboratory would be part of HIVST regulation but the ZAMRA were considering outsourcing to a different laboratory: “The best outsourced reference lab that I might point out is the Bureau of Standards” (Zambia KII6 Female).

Memoranda of understanding were identified as a possible mechanism by which different organizations could work together. However, regulatory key informants in Zimbabwe thought split mandates needed to be addressed in a way that the mandate rests with one regulator only: “How we team up with them is through MOUs” (Zimbabwe KII10 Female). “So, let's work together you as medicine laboratory scientists, evaluating controlling regulating kits for us but not the other way around” (Zimbabwe KII27 Female).

### 3.3 International and regional harmonization

WHO pre‐qualification was recognized by key informants from all sectors, across all countries, as an important mechanism for ensuring the quality of test kits from manufacturers. Most also stated that it was a procurement requirement from donors as illustrated by this commonly held view: “For now, we are happy to look at what WHO has recommended as a bare minimum, then we will add additional prerequisites ourselves, but it must have a recommendation from WHO. If they are fully pre‐qualified that's even better” (Zambia KII12 Female).

There was little mention of the existence of any regional bodies or other inter‐country interactions other than with the WHO for IVD regulation, but participants from all sectors were aware of the benefits of a shared approach and were open to the possibility. One regulatory key informant indicated they were using other countries’ regulations to base their own draft IVD regulations on: “you see, we pick it up from different countries and then we sort of custom make our own” (Zimbabwe KII10 Female). Similarly, ZAMRA were reported to be looking at regional collaboration: “We will sit down as regulators and say fine how are we going to look at this because I know Zimbabwe had some guidelines” (Zambia KII6 Female). Recognition of regional efforts for collaboration was also seen amongst laboratorians and regulators as indicated by this key informant: “It's a matter of trying to get Malawi at the table to see how other countries are doing so they can set up something similar” (Malawi KII13 Male).

## 4 Discussion

HIVST is a relatively new technology, especially in the context of regulations in low‐ and middle‐income countries 27. Our research found that the development of regulation for IVDs ranged from none in one country to the drafting of guidelines for pre‐market regulation in the other two countries. We found lack of clarity of roles and responsibilities across different organizations and regulatory authorities making it difficult to determine who was responsible for HIVST regulations in country. We also found that overlapping mandates for regulating *in vitro* diagnostics may be a significant factor in delaying the development of regulations and could result in stalemate or the development of conflicting regulations. Key informants we interviewed were particularly concerned about the performance of HIVST in the hands of intended users and the implications for post‐market surveillance despite evidence to the contrary 28.

The potential role of HIV National Reference Laboratories who are already monitoring HIV kits for professional use, in post‐market surveillance of HIVST had not been recognized by most regulators. There is a clear, recognized need for strengthened regulatory capacity for medical device regulators, HIV departments and National Reference Laboratories and clarity on their roles in HIVST regulation, so policy and regulation can be properly aligned and the experience of reference laboratories in checking HIV kits can be properly utilized.

HIVST regulation and implementation is a rapidly evolving field. A study conducted in 2013 involving similar constituents, and in the case of Malawi some of the same individuals, showed that few participants had come across HIVST in practice 29. Concerns were voiced about the need for counselling and the potential for coercive testing and to a lesser extent about kit accuracy. In contrast, we found widespread familiarity with HIVST as an approach and more focus on concerns over test performance and systems for quality assurance. The development of post‐market surveillance systems able to detect false non‐reactive results is of concern in other studies too 29, 30 but we are not aware of any programmes that have successfully addressed this. Current HIV quality assurance approaches are designed for facility‐based rapid testing, where testing is conducted by trained testers who record results and where kit storage and lot numbers can be traced 31. Alternative approaches to monitoring HIVST performance, such as the visual stability of kits for re‐reading, digital photography and direct observation need further investigation 32, 33.

HIVST regulation has failed to keep pace with the scale‐up of HIVST and IVD regulation in general and is underdeveloped in many countries 11, 12. Globally, only one HIVST device has been pre‐qualified by the WHO and the process to gather the evidence required for dossier submission can take many years 34. Our findings of poor national‐level coordination and capacity have implications for both manufacturers trying to enter these local markets and for end users. For manufacturers, the fragmented and uncertain regulatory environment creates barriers that mean they are reluctant to take the financial risks associated with the development of high quality HIVST products. Manufacturers lack incentives to innovate further product development and prices for existing products remain high due to lack of competition 35. For end users, the delays could result in the proliferation and use of unregulated low quality tests and ultimately incorrect HIV screening results and loss of consumer confidence 36.

The current lack of IVD regulation in many African countries presents an opportunity for regulatory convergence between countries. Regional groups, such as the Pan African Harmonization Working Party 37 and the African Society for Laboratory Medicine 38, already exist with this aim but lack adequate resourcing and political prioritization. Regional coordination can develop capacity, save time and effort and speed up costly, cumbersome and duplicative processes. Four key areas to aid convergence are: common pre‐market registration; joint manufacturing site inspections; joint data review and evaluation protocols and the establishment of laboratory networks for post‐market surveillance 12, 39. Our findings indicate WHO pre‐qualification is likely to be an important component of any common pre‐market HIVST registration system in the African region provided manufactures buy into the process 40. Collaborative regulatory procedures (e.g. reviews of dossier submissions) make a more attractive regulatory environment for manufacturers who would no longer need to submit different dossiers to multiple regulatory authorities 41.

National leadership links HIVST to the wider HIV testing strategy and brings key stakeholders under a common vision. A single coordinated approach that establishes roles and responsibilities from an early stage, will allow a complete picture of HIVST situation within the country, linking HIV testing policymakers, regulators and laboratory stakeholders. Experience from policy development elsewhere reveals that power, inclusive of funders, politics and patronage can play as much of a role in delaying or pushing through policy as evidence and need 42. The current fragmented approach risks exacerbating this situation when there is a lack of direction, convergence within intra‐country constituencies and strong leadership.

The study shares limitations of qualitative approaches in general, principally the non‐generalizability of study findings. However, the qualitative approach insisting on depth rather than breadth was suitable for our study since it enabled us to explore, describe and analyse sensitive issues related to a new testing approach. Though we interviewed a wide range of participants across seven different sectors, the small number of respondents in some categories made comparison across groups difficult. Due to a limited number of possible respondents, countries rather than constituencies have been used for attribution of illustrative quotations to protect individual's anonymity. Some areas related to regulation such as government procurement processes and supply chain were not explored in depth during the interviews to try and focus more on the barriers and opportunities to developing regulations.

## 5 Conclusions

The recognition of the role of regulation in the scale‐up of HIVST is important to ensure the market only has high quality test kits that can be used correctly and confidently by intended users. Programmes should establish clear lines of communication with IVD regulators early to allow for the alignment of policy and regulation and ensure all voices are heard in their respective development. The expertise of HIV National Reference Laboratories should be used to assist in the evaluation of HIVST kits and the development of post‐market surveillance systems.

## Competing interests

None of the authors have any conflicts of interest.

## Authors’ contributions

RD was involved in the concept, design, data collection and management, analysis of study data and led the writing of the manuscript. VW was involved in the concept, design, data collection and management of the study and contributed to the writing of the manuscript. LN was involved in the design and data collection portion of the study and contributed to the writing of the manuscript. CM, LC ad MS were involved in the data collection portion of the study and reviewed the manuscript. CJ, ELC and KH were involved in the concept and design of the study and reviewed the manuscript. MT was involved in the concept, design and analysis of the study and contributed to the writing of the manuscript.

## Supporting information


**Data S1.** Interview Guide for Key National Stakeholders: HIVST regulatory and policy.
