# Supplementary material for: Regulation of HIV self‐testing in Malawi, Zambia and Zimbabwe: a qualitative study with key stakeholders
Source: J Int AIDS Soc. 2019 Mar 25;22(Suppl Suppl 1):e25229. doi: 10.1002/jia2.25229 (PMC6432109; doi:10.1002/jia2.25229)
Supplement: Supplementary file 1 — Data S1. Interview Guide for Key National Stakeholders: HIVST regulatory and policy. [file JIA2-22-e25229-s001.docx]

**Supplementary File 1: Interview Guide for Key National Stakeholders: HIVST regulatory and policy**

**Definition key stakeholder:** government officials involved in HIV control/MOH, national reference lab, medical regulatory authority, Bureau of Standards, regulatory lawyers, national medical stores, procurement agents, donors, academics, major implementing partners (including NGOs).

*OBTAIN INFORMED CONSENT*

**General information**

1. What is your professional designation?
2. What is your role in HIV services and/or management?
3. How long have you been working in HIV services and/or management?
4. What do you know about the different approaches to HIV self testing?

**CONTENT**

1. What do you know about the current polices and guidelines in place for HIV self-testing?

PROBES: Is there a *specific* policy addressing HIV self-testing? Does it distinguish between different samples used of self testing (i.e. blood and saliva)? Is self-testing mentioned in the national HIV guidelines or strategic plans? If current policy is absent or does not have much detail, why do you think that is? what do you think would need to be changed to allow HIV self-testing in policy?

1. What are the main aspects of current policy that support HIVST?
2. What are the main policy gaps for HIV self-testing?

PROBES: probe to explore if they cover areas such as HIVST testing algorithms, protocols for confirmatory testing, linkage to care, procurement and distribution of kits? Do they restrict who is allowed to perform HIV testing? Are there any additional documents being planned or in draft form?

*Counselling, consent and confidentiality in policy*

1. Should counselling accompany HIVST?
2. Is there any policy or guidance on consent for HIV testing?

PROBES: What are the main issues of consent in HIVST in your opinion? Do you think consent has been given adequate consideration? Are there any groups who would be difficult to manage with regards consent (e.g. adolescents)? Are there mechanisms in place to allow testing of these groups (e.g. consent from guardian)? Are there any groups in which legal consent is not required for HIV self-testing (e.g. prisoners)?

1. Are there debates or concerns that HIV self-testing has potential for coercion? Might it be used for coercive testing?

PROBES: Do you think there are situations in which HIV ST could be used to discriminate against those with HIV (e.g. workplace, insurance)

1. Do you think confidentiality is an issue when applied to HIV self-testing? If yes how?

*Linkage to care in policy*

1. How can HIV self-testing be most effectively linked to care?

**ACTORS**

1. Who are the key stakeholders HIVST? What are their roles?

PROBES: These can be individuals or organisations. Why are these people/organisations important? Who supports HIVST? Who has concerns over HIVST?

1. Do you see any changes in attitudes toward HIV self-testing in the last year?

PROBE: If so, who has been mainly responsible for these changes?

*Actors in regulation*

1. What official bodies in [insert country] would be responsible for the regulation of self-testing and self-testing kits for HIV?

PROBE: Which of these bodies are the most critical? Within those bodies, who would be the most involved?

1. What regional and international regulatory groups are you aware of? What, if any, is their role in the implementation of HIV self-testing in [insert country]?
2. How do you think regulatory bodies and professional laboratory groups would react to the introduction of HIV self testing?

PROBE: Ask about contacts in these groups and any legal regulatory contacts.

*Actors in monitoring*

1. Which different interest groups should be included in monitoring HIV self testing?

PROBE: Can you think if any groups in the following sectors: legal, human rights, PLHIV, laboratory, Government Ministries, NGOs, donors, universities, regulatory authorities.

1. Is there a taskforce for HIV testing and counseling?

PROBE: Is there a group tasked with looking at HIVST issues in specific?

1. Can you provide the names of the people who best represent these interest groups so we can include them in the discussion and possibly interview them?

**CONTEXT**

1. Are HIV self-tests and self-testing currently regulated in [insert country]?

PROBE: Please explain what you know about their current legal and regulation status. Does it distinguish between different sample types (i.e. blood and saliva)?

1. If HIV self-tests or HIV self-testing are not currently regulated do you know of any plans to subject them to regulation?
2. Is there any policy or guidance on who can import, sell or distribute HIV tests?

PROBE: Do you think these guidelines are also appropriate for HIV self-tests? Do you think distribution and selling of HIV self-tests require any special considerations?

1. Are there registration and certification requirements for vendors, distributors and manufacturers for the legal purchase and use of HIV self-tests by consumers?
2. Are there any barriers to selling HIV self-tests or distributing them for free?

Is it possible that manufacturers and distributors could be legally liable for HIV self-testing (e.g. test causes harm)? How?

PROBES: What legal and regulatory protections are in place to address potential harms associated with incorrect results? Do you think these are sufficient? If not what else would be required to be put in place?

1. How do you think the advertising of HIVST should be approached in [insert country]

PROBES: What barriers might there be to advertising using branded products. What organizations would need to be involved? What do you think are the main targets of messaging for HIVST and how could they be best reached?

**PROCESS**

1. How has HIVST happened in [insert country] to date? What have been the steps so far?

PROBES: What have been the main barriers to HIV self-testing? How could these barriers best be overcome?

1. How ready to you think [insert country] now is for scale-up of HIV self-testing?

*Monitoring*

1. How can the accuracy and safety of HIVST be monitored?

PROBE: This includes they type of sample used, monitoring the quality of the tests and the ability of users to obtain an accurate result. (FOR LAB PEOPLE: How would you go about designing an EQA system for HIVST for both blood and saliva? How useful would re-reading of tests and/or videos of testing be?). What opportunities would there be to test EQA models?

1. What are the challenges with monitoring HIVST?

PROBE: Can you see any solutions to overcome these challenges?

1. Are any other devices used for self- or home-testing (e.g. pregnancy tests, malaria RDTs)?

PROBE: What lessons can be learned from monitoring these?

1. How do you think the link between testing and seeking care could be monitored?

*Next steps*

1. What next steps are needed to scale-up of HIV self-testing?

PROBE: What are the mechanisms for changing policy in relation to HIV testing and counseling? What else is required in terms of regulation? Logistics? Capacity strengthening to scale up HIV self-testing?

1. What would your role be in the scale-up of HIV self testing?

PROBE: How would you interact with other involved in this process to fulfill your role?

1. Do you have any other comments

Thank you
